# Enhancing adverse drug reaction data quality in Canada: A high-precision pipeline for medication name standardization and enrichment

**DOI:** 10.1371/journal.pone.0331940

**Published:** 2025-09-25

**Authors:** Niaz Chalabianloo, Sheikh S. Abdullah, Mohammad Ali Omrani, Atefeh Jafari, Kamran Sedig, Flory Tsobo Muanda

**Affiliations:** 1 Department of Physiology and Pharmacology, Western University, London, Ontario, Canada; 2 Department of Computer Science, Western University, London, Ontario, Canada; 3 ICES Western, London, Ontario, Canada; 4 Department of Computer Science, MacEwan University, Edmonton, Alberta, Canada; 5 London Health Sciences Centre Research Institute, London, Ontario, Canada; 6 Department of Epidemiology and Biostatistics, Western University, London, Ontario, Canada; 7 Faculty of Information and Media Studies, Western University, London, Ontario, Canada; 8 Lawson Health Research Institute, London Health Sciences Centre, London, Ontario, Canada; University of Science and Technology of Fujairah, YEMEN

## Abstract

**Background:** The Canada Vigilance Adverse Reaction database is a vital pharmacovigilance tool, but its utility is severely limited by heterogeneity in medication nomenclature. A substantial portion (∼36.8%) of unique drug name variants in the database lack any mapping to an active ingredient, representing a critical data quality gap that can mask important adverse drug reaction (ADR) signals.

**Methods:** We developed, validated, and publicly released a high-precision, automated pipeline to standardize and enrich medication names. The pipeline employs a cascaded matching strategy that leverages the RxNorm and Observational Health Data Sciences and Informatics (OHDSI) vocabularies. Standardized names are assigned a RxNorm Concept Unique Identifier (RxCUI) and enriched with active ingredient data and Anatomical Therapeutic Chemical (ATC) classifications via RxNav APIs. The pipeline’s accuracy was rigorously assessed by two independent experts on a balanced validation set of 200 cases.

**Results:** The final pipeline successfully standardized 94.5% of the 46,585 unique drug names. A blinded expert validation confirmed high reliability, demonstrating a precision of 98.02% (95% CI: 0.9307–0.9946) and specificity of 97.22% (95% CI: 0.9043–0.9923). Case studies showed that standardization and aggregation of reports revealed known safety signals (e.g., mesalamine and asthenia) that were statistically undetectable in the raw data.

**Conclusion:** Our transparent and reproducible pipeline effectively resolves medication name heterogeneity in Canada’s national ADR database. By transforming variable text into standardized concepts, it significantly enhances data quality, improves the sensitivity of safety signal detection, and facilitates interoperability with global health datasets. The publicly available tool provides a valuable resource for strengthening drug safety surveillance in Canada and beyond.

## Introduction

The Canada Vigilance Adverse Reaction database is a cornerstone of Canada’s post-market drug surveillance system, accumulating reports of suspected adverse reactions since 1965 [1,2]. While invaluable for pharmacovigilance, its utility is significantly hampered by severe heterogeneity in medication nomenclature. Reporters use varied brand names, generic names, abbreviations, and misspellings, fragmenting the data and impeding aggregation, data linkage, and reliable adverse event signal detection [3]. Adverse events linked to a single drug may be scattered across numerous name variants, potentially falling below statistical detection thresholds.

**Fig 1:**
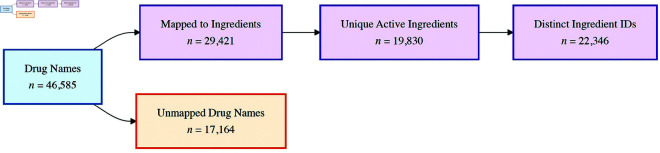
Hierarchical structure of drug name heterogeneity in the Canada vigilance database. The diagram illustrates the substantial nomenclature fragmentation observed.

This nomenclature challenge is substantial. Analysis of the database (Fig 1) reveals 46,585 distinct drug names. Of these, 29,421 names have established mappings, while a critical 17,164 drug name variants (∼36.85%) appear in reports without any ingredient linkage, representing a significant analytical blind spot. These unmapped names often involve combination products (16.7% contain indicators like “with,” “plus,” or “/”) or non-standard terminology (e.g., “PILLS FOR GAS,” or truncated names). The mapped names correspond to 19,830 unique active ingredients, but heterogeneity persists even here. This is evidenced by the fact that these ingredients are tracked using 22,346 distinct internal identifiers, indicating that a single substance is often referenced by multiple codes. For instance, acetylsalicylic acid appears under 149 textual variations associated with 164 distinct ingredient identifiers, and 2,303 ingredient names ambiguously reference multiple chemical entities.

Standardizing medication names in pharmacovigilance databases is a well-recognized challenge, and various computational strategies have been developed to address it. Common approaches start with exact string matching against reference lists after basic text cleaning (lowercasing, removing punctuation). However, exact matching, while precise, often suffers from low recall when faced with misspellings or minor variations [10]. To improve coverage, pipelines frequently incorporate heuristic rules, such as stripping dosage forms, routes, and strength information to isolate the core drug name. These rule-based systems alone struggle with typos and subtle name differences, which has led to the adoption of approximate (“fuzzy”) string matching algorithms. By measuring string similarity, fuzzy matching can identify likely standard names despite errors, significantly improving mapping rates with minimal manual effort [11,12].

Effective standardization also relies on robust reference terminologies. RxNorm, developed by the U.S. National Library of Medicine, provides a normalized naming system linking various drug vocabularies via unique identifiers (RxCUIs), facilitating mapping between brand names, ingredients, and clinical formulations [11]. The WHODrug Global dictionary offers a complementary international standard, assigning codes and Anatomical Therapeutic Chemical (ATC) classifications to medications [13]. Internationally, these tools have been used to great effect. Researchers have normalized thousands of name variants in the FDA’s FAERS database using the RxNorm API [14], and WHO’s VigiBase employs an AI-assisted engine that achieves high accuracy (∼97%) and automated coding rates (∼89%) for millions of drug entries [15].

In stark contrast, Canada Vigilance currently lacks a structured medication name standardization framework [2]. While global initiatives increasingly standardize ADR data to improve signal detection and interoperability [4–7], the public data from Canada Vigilance provides only verbatim names that require external standardization by researchers [3]. This gap hinders the application of advanced analytical methods [8,9], limits the integration of Canadian data into global pharmacovigilance efforts, and underscores the need for a tailored standardization pipeline.

This study aimed to develop and refine a rigorous, iterative pipeline for medication name standardization within the Canada Vigilance database. Our specific objectives were:

- Incrementally increase the coverage and accuracy of matching reported drug names to a standard vocabulary (RxNorm with ATC linkage) through successive algorithmic enhancements;
- Evaluate the impact of these enhancements quantitatively, qualitatively, and through expert validation;
- Examine the implications of the standardized dataset on pharmacovigilance analyses;
- Demonstrate potential interoperability with international databases; and
- Ensure transparency and reproducibility by documenting the implementation and sharing the code.

## Methods

To address the significant medication nomenclature heterogeneity within the Canada Vigilance database (see the Introduction section, and Fig 1), including inconsistencies at both brand and ingredient levels, we developed and iteratively refined a multi-stage standardization pipeline. The primary goal was to reliably map the diverse reported drug names to standardized RxNorm concepts and enrich these mappings with relevant classifications (e.g., ATC) suitable for downstream analysis. We applied the pipeline to all 46,585 distinct drug name entries identified, crucially including the 17,164 variants lacking prior ingredient mappings. These unmapped cases often represented challenging instances such as combination products or non-standard terms.

Although 29,421 of these names had pre-existing mappings to an active ingredient field in the source data, this field itself contained significant heterogeneity and did not use a standard controlled vocabulary. Therefore, applying our pipeline to all 46,585 distinct names was essential to ensure every term was consistently mapped to a single, authoritative reference terminology (RxNorm) and enriched with standardized ATC codes, creating a truly harmonized dataset.

### Overall architecture

**Fig 2:**
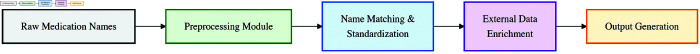
High-level pipeline overview. A schematic representation of the main components of the medication name standardization pipeline.

The final version of our pipeline (v9) employs a modular, sequential architecture designed for robustness, accuracy, and maintainability (Fig 2). Raw medication names pass through dedicated modules for preprocessing, name matching/standardization, external data enrichment, and output generation. A core design principle was to leverage established, high-quality external knowledge bases (RxNorm, and OHDSI (Observational Health Data Sciences and Informatics) vocabularies accessed via APIs) rather than rely solely on local dictionaries or complex custom rules. This approach benefits from curated terminologies and reduces the local maintenance burden.

A multi-tiered caching system, using separate JSON files for different processing stages (preprocessing results, RxNorm/OHDSI lookup results, and final enrichment data), is critical for performance and resilience. Caching minimizes redundant external API calls, respects API rate limits, and allows the pipeline to effectively resume processing after interruptions without losing progress on completed items. We implemented comprehensive caching not only for performance optimization but also to ensure robustness against transient network issues or API downtime during large-scale processing runs. (Detailed descriptions of module internals, specific algorithms, and caching architecture are provided in S1 Text.)

### Key pipeline stages

The pipeline consists of the following sequential stages:

#### Preprocessing.

This initial stage aims to normalize raw drug names and reduce noise that can hinder matching accuracy. Standard transformations are applied, such as converting text to lowercase and removing punctuations. Critically, the preprocessing module systematically removes a wide range of non-essential terms using a curated list (unwanted_terms.txt). This includes dosage forms (e.g., ‘tablet’, ‘injection’), strengths and units (e.g., ‘mg’, ‘ml’), routes of administration (e.g., ‘oral’, ‘IV’), packaging information, manufacturer names, and common filler words (e.g., ‘and’, ‘with’, ‘plus’). Common abbreviations (for example, ‘HCL’ is expanded to ‘hydrochloride’) are also expanded, and simple delimiters like ‘\ /’ are replaced with spaces to aid tokenization. The goal of these steps is to isolate the core pharmacological concept(s).

Given the bilingual context of the Canada Vigilance database, specific support for French-language terms was also incorporated into the preprocessing stage. This was achieved through two primary mechanisms: first, by including common French pharmaceutical terms (e.g., comprimé for tablet, gouttes for drops) in the ‘unwanted_terms.txt’ list for removal during normalization; and second, by adding direct mappings for specific French brand or generic names to their English-equivalent standardized concepts within the ‘special_cases_canada.txt’ file. While not exhaustive, this targeted approach improves the handling of common bilingual variations found in the data, and its limitations are further discussed in the ‘Limitations and Future Directions’ section.

While our pipeline can standardize some combination products by cleaning delimiters (e.g., ‘/’), and the subsequent RxNorm API step may handle common phrasings implicitly [10,16], it does not implement a generalized parser to systematically deconstruct complex multi-ingredient names that lack pre-coordinated concepts in RxNorm.

To address this and other challenging cases, we handle specific known variants via targeted rules defined in ‘special_cases.txt‘. This curated list was developed through an iterative, manual analysis of terms where the automated pipeline initially failed, produced an ambiguous match, or for which a direct local mapping was determined to be significantly more efficient. Each rule in this file represents a high-confidence mapping, often confirmed through domain expertise, designed to address several specific challenges:

- **Resolving Ambiguity:** It provides a definitive mapping for terms known to cause ambiguous or incorrect matches with purely algorithmic approaches.
- **Mapping Complex and International Names:** It ensures accurate standardization for specific multi-ingredient products (e.g., mapping the supplement metanx to its components) or international brand names (e.g., doliprane to Paracetamol) that are not present in the core RxNorm terminology.
- **Handling Coded and High-Frequency Terms:** It guarantees the correct and efficient standardization of terms that are not discoverable via standard search, such as research codes (e.g., lcz696 to Sacubitril / Valsartan), or other high-frequency non-standard phrases.

This targeted, knowledge-driven approach complements the generalized matching capabilities of the downstream modules. While this list is extensive, it represents a small fraction of the total unique names processed, and its modular design allows for future additions as new challenging terms are identified. (See Table S2 in S1 Text for the full list of challenge categories and examples.)

#### Name matching strategy.

**Fig 3:**
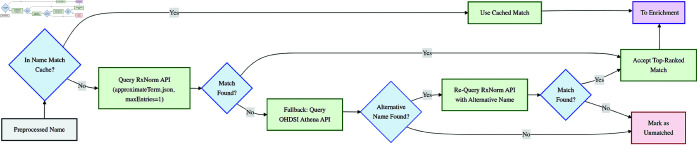
Name matching strategy. Decision flow diagram for the name matching process, illustrating the cascade of matching techniques and fallback mechanisms.

We employed a cascaded strategy (illustrated in Fig 3) to efficiently find a standardized RxNorm Concept Unique Identifier (RxCUI). This approach prioritizes accuracy and leverages external intelligence through the following steps:

1. **Cache Check:** Avoid reprocessing by retrieving previously successful matches from the cache.
2. **Primary RxNorm Query and Evaluation:** If no cache entry exists, submit the term to the RxNorm approximateTerm.json API endpoint, requesting only the single best match (maxEntries=1) [10,16]. This API uses RxNorm’s extensive internal synonymy, lexical variant generation, and term relationships to identify the most relevant standardized concept (RxCUI), effectively handling both exact matches and variations like misspellings.
3. **Match Acceptance:** If the RxNorm API returns a candidate match (i.e., the query is successful and a relevant concept is found), accept this top-ranked match (identified by its RxCUI and standardized name) without further evaluation. This corresponds to the successful match outcomes after the API query as depicted in Fig 3.
4. **Fallback OHDSI Lookup:** If the primary RxNorm query (Step 2) fails to return any candidate match (e.g., the term not recognized by RxNorm, potentially due to being a non-US product), then query the OHDSI Athena API as a fallback. This API searches a broader set of vocabularies (including RxNorm Extension) for a potential match. We parse the Athena API’s JSON response to extract a plausible alternative name or identifier if available, aiming to identify international or alternative representations of the original term.
5. **Secondary RxNorm Query:** If a plausible alternative name is obtained from OHDSI, resubmit that candidate name to the RxNorm approximateTerm.json API (maxEntries=1) as a final attempt to anchor the term in the RxNorm vocabulary. If this secondary query returns a match, accept it as the standardized result.

This multi-step approach relies on RxNorm’s ability to provide the single most appropriate match (exact or approximate) and uses a fallback mechanism via OHDSI only when the primary RxNorm search yields no result. Any name that remains unresolved after both the primary and secondary RxNorm is marked as unmatched. (Detailed logic and error-handling nuances are provided in S1 Text.)

#### External data enrichment.

**Fig 4:**
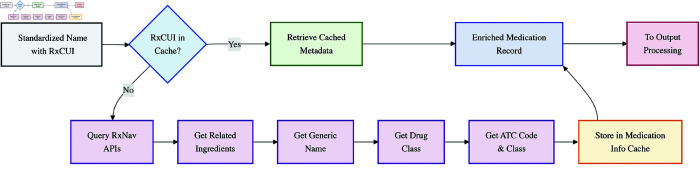
External data enrichment module. Workflow diagram showing the sequential retrieval of medication metadata from RxNav APIs.

Once an RxCUI is successfully assigned, the pipeline enriches the record with additional information retrieved exclusively via various RxNav APIs (Fig 4). This ensures that all supplementary details (ingredients, ATC codes, drug classes) are directly and authoritatively linked to the identified RxNorm concept. Key enrichments include:

- **Related Ingredients:** The active ingredient(s) name(s) and corresponding RxCUIs (queried via RxNav’s related.json?tty=IN endpoint for ingredients).
- **Standardized Generic Name:** A standardized generic name representation for the drug (obtained via RxNav; for branded products, this is typically the name(s) of the active ingredient(s), while for generic concepts, it is the normalized RxNorm name).
- **Drug Class information:** Relevant drug class data (via the RxNav RxClass API, such as Established Pharmacologic Class).
- **ATC classification:** The Anatomical Therapeutic Chemical code and class name (via RxNav’s RxClass API, typically linked to the drug’s ingredient concept).

(See S1 Text for specific API calls and detailed enrichment procedures).

#### Output generation.

The final stage consolidates the standardized results. A mapping resource is created, linking original drug names to their standardized RxCUI, name, ingredients, ATC codes, and other retrieved metadata. This output is structured (e.g., CSV file) for merging back with the original ADR report data for subsequent analysis. (Further output processing details can be found in S1 Text.)

### Iterative refinement and justification

The final v9 pipeline represents the culmination of an iterative development process. Early prototype versions were significantly improved through quantitative performance evaluation at each stage. Enhancements included refining preprocessing rules, transitioning from simple exact matching to using the RxNorm approximate match API, strategically incorporating the OHDSI fallback, adding ATC enrichment via RxNav, implementing robust caching, and introducing support for French-language terms common in Canadian data. This iterative refinement, with performance evaluated at each stage, was essential for achieving the final high match rates and accuracy. The quantitative outcomes of these iterations are presented in the Results section (Table 1), and full details of the development history are in S1 Text.

**Table 1:** Match rates by major iteration of the standardization pipeline.

Iteration (Version)	Key Enhancements	Matched Names (%)
v1 (Baseline)	Basic RxNorm lookup (no preprocessing, no Fuzzy matching)	58.7%
v2	+ Simple preprocessing (term removal)	65.4%
v3	+ Fuzzy matching for misspellings	75.1%
v4	+ OHDSI & synonym integration	82.3%
v5	+ Caching (efficiency improvement)	82.5%
v6	+ Added ATC enrichment	82.5%
v7	+ Robustness fixes (edge cases)	85.0%
v8	+ Multilingual (French) handling	87.2%
v9 (Final)	+ Final tweaks & manual special cases	94.5%

### Data source

We evaluated the standardization pipeline’s performance using the complete set of 46,585 distinct medication names extracted from the Canada Vigilance database (data spanning 1965-2024). The data extract from the Canada Vigilance database was accessed for this research in February 2025.

### Performance evaluation and validation

We evaluated the pipeline’s performance through a multi-faceted approach. We focused on improvements in match rates across development iterations, final performance metrics, qualitative outcomes, expert validation, and the pipeline’s impact on signal detection.

#### Quantitative performance metrics.

The primary quantitative performance metric was the Match Rate, defined as the percentage of unique medication names successfully mapped to a non-null RxNorm Concept Unique Identifier (RxCUI). We tracked this metric across pipeline versions to evaluate the impact of iterative enhancements (Table 1). We also measured Enrichment Success by calculating the proportion of matched entries for which we could retrieve ingredient information and Anatomical Therapeutic Chemical (ATC) classifications. Additionally, we monitored computational performance by measuring the processing time for a sample of uncached terms.

#### Expert validation.

To rigorously assess the algorithm’s accuracy beyond automated metrics, we conducted a human-in-the-loop validation. While the operational dataset is highly skewed towards successful matches (over 94%), a balanced validation set was intentionally constructed, comprising 200 medication name pairs derived from the pipeline’s output. This set included 101 pairs where our algorithm found a match (’Match Found’) and 99 pairs where it did not (’No Match Found’). This case-control design, a standard approach for evaluating classifiers on imbalanced data, was chosen to ensure that a sufficient number of both positive and negative cases were included. This allows for the robust estimation of key performance metrics such as specificity and recall, which would be statistically unstable if evaluated on a small number of cases drawn from a prevalence-based sample.

The total sample size of 200 was selected as a pragmatic balance between the intensive manual labor required for dual-expert review and the need to achieve stable estimates for our primary accuracy metrics. As the primary goal of this validation was to *estimate* the pipeline’s performance metrics (e.g., precision, specificity) rather than to test a formal statistical hypothesis of effect, a formal *a priori* sample size calculation based on statistical power was not performed. This approach aligns with modern standards for diagnostic accuracy and model validation studies, where the objective is quantification and precision rather than null-hypothesis testing [17–19]. The statistical precision achieved with our sample size is, therefore, transparently reported using 95% confidence intervals for all key performance metrics, which is the recommended practice for communicating the certainty of performance estimates in such studies [20,21].

Two independent domain experts (pharmacists with pharmacovigilance and nomenclature experience) reviewed each medication name pair, determining whether the original and standardized names represented the same medication entity. The experts were blinded to the algorithm’s decisions to prevent bias. We defined expert consensus as cases where both experts agreed on a “YES” judgment for a pair; this consensus served as the reference standard for our evaluation.

#### Impact on signal detection analysis.

To demonstrate the practical impact of standardization, we conducted illustrative case studies on known drug-event associations. We performed a disproportionality analysis on the Canada Vigilance data both before and after applying our standardization pipeline. We calculated standard pharmacovigilance metrics, including the Information Component (IC) and the Reporting Odds Ratio (ROR), using a report-based unit of analysis. This approach was chosen for its robustness to variations in report complexity present in the source data. A signal of disproportionate reporting (SDR) was considered present if the lower bound of the 95% credibility/confidence interval was greater than the threshold (IC025 > 0 or ROR lower bound > 1).

(Detailed methods for signal calculation, the rationale for using a report-based approach given the data characteristics, and further analysis of report complexity reduction are provided in S1 Text.)

### Ethics statement

This study utilized publicly available, de-identified data from the Canada Vigilance Adverse Reaction Database, obtained from the official Government of Canada open data portal. The research team did not have access to any identifying patient information. As the data are anonymized and publicly accessible, this research did not require review or approval from an institutional review board.

## Results

### Performance and standardization outcomes

The iterative refinement process resulted in dramatic performance enhancements across nine major versions of the pipeline. As summarized in Table 1, the match rate increased from a baseline of 58.7% to a final 94.5%. This was achieved through successive enhancements including advanced preprocessing, the use of RxNorm’s approximate matching API, incorporation of an OHDSI fallback for international names, support for multilingual terms, and comprehensive caching. Ultimately, the final pipeline version (v9) successfully standardized 44,034 out of 46,585 distinct drug name entries. (Full details of the development history are provided in S1 Text).

Qualitative review confirmed the pipeline’s ability to correctly handle a wide range of cases (Table 2). It accurately performed brand-to-generic mappings, corrected common misspellings, recognized combination products and mapped them to appropriate standardized concepts, and dealt with multilingual name variants common in Canada.

**Table 2:** Examples of medication name standardization outcomes.

Raw Reported Name	Preprocessed Name	Standardized Name (RxNorm)	Notes
TYLENOL 500 MG CAPLETS	tylenol	Acetaminophen	Brand to ingredient mapping; dose form removed.
Humira (no strength given)	humira	Adalimumab	Brand to ingredient (biologic).
Metformn (misspelled)	metformn	Metformin	Approximate matching corrected spelling.
Losartan/HCTZ 50/12.5	losartan hctz	Losartan / Hydrochlorothiazide	Combination product recognized and mapped to a pre-coordinated concept.
Doliprane (*International Brand*)	doliprane	Paracetamol	International brand handled via curated ‘special_cases.txt‘ file.
???? (garbled entry)	(none)	Unmatched	Non-informative name correctly remains unmatched.
ACCU-CHEK AVIVA PLUS (device)	accu chek aviva plus	Unmatched (device)	Medical device correctly identified and not matched to a drug concept.

### Enrichment success

Beyond accurate name standardization, the pipeline also enriched the matched entries using RxNav APIs, adding valuable pharmacological context. Ingredient mapping (linking the matched RxCUI to its corresponding ingredient RxCUI(s)) was achieved for over 98.6% of the matched names. Furthermore, Anatomical Therapeutic Chemical (ATC) classification codes (typically 4th-level ATC codes) were retrieved via RxNav’s RxClass API for approximately 73.8% of matched entries. (Enrichment process details are available in S1 Text.)

### Computational performance

A performance benchmark was established by executing the pipeline on the full set of unique medication names. The process was run on a MacBook Pro computer (Apple M3 Pro processor, 36 GB RAM) with a 1 Gbps synchronous internet connection. The total execution time for an initial, uncached run of all 46,586 unique terms was approximately 3 hours and 46 minutes (226 minutes). The majority of this time was consumed by network latency from the sequential API calls required for the matching and enrichment stages. Subsequent runs on the same data are near-instantaneous due to the comprehensive caching mechanism, demonstrating the critical role of the caching system in optimizing performance for practical use.

### Expert validation

Inter-rater reliability between the experts was strong (Cohen’s kappa (κ)= 0.8405), indicating consistent human judgment. Compared against expert consensus, the algorithm demonstrated exceptional reliability, as summarized in Table 3.

**Table 3:** Expert validation performance metrics of the standardization pipeline.

Performance Metric	Value	95% Confidence Interval
Precision	0.9802	(0.9307 – 0.9946)
Specificity	0.9722	(0.9043 – 0.9923)
Recall (Sensitivity)	0.7734	(0.6936 – 0.8374)
**Performance Metric**	**Value**	
Balanced Accuracy	0.8728	
Matthews Corr. Coeff. (MCC)	0.7159	
Inter-Rater Kappa (κ) (Experts)	0.8405	

The pipeline achieved a precision of 98.02% (95% CI: 0.9307 – 0.9946) and a specificity of 97.22% (95% CI: 0.9043 – 0.9923). This indicates the pipeline rarely produces incorrect matches and correctly identifies true non-matches. The recall was 77.34% (95% CI: 0.6936 – 0.8374), reflecting a deliberately conservative approach that prioritizes high precision. The strong balanced performance (Balanced Accuracy: 0.8728; MCC: 0.7159) further confirms the pipeline’s effectiveness across both matched and unmatched cases.

Error analysis showed that false positives were extremely rare (1.98%) and typically involved very closely related drugs. False negatives often involved complex synonymy (e.g., “Aspirin” vs. “Acetylsalicylic acid”), international naming variants (e.g., “Paracetamol” vs “Acetaminophen”), or uncaptured abbreviations.

### Analysis of unmatched names

To understand the reasons for matching failure, we performed a systematic characterization of the 2,551 (∼5.5%) unmatched terms. We first applied a programmatic analysis using keyword and pattern matching, which successfully categorized 261 terms into unambiguous groups such as medical devices or research codes. A qualitative manual review was then conducted on the remaining 2,290 terms to identify the primary failure themes. The results of this hybrid analysis are summarized in Table 4. The characterization revealed that the majority of unmatched terms were not simple pharmaceuticals, but rather Natural Health Products, medical devices, vague descriptions, or international brands not found in the core terminologies. Crucially, we also identified a small subset of “Pipeline Misses”—terms like simple misspellings or known drugs that the pipeline failed to standardize, highlighting the inherent fallibility of any fully automated process.

**Table 4:** Systematic characterization of the 2,551 unmatched medication name entries.

Primary Reason for Matching Failure	Illustrative Example(s)
*Programmatically Identified Categories (n=261)*	
Medical Device or Equipment	AEROCHAMBER, PACEMAKER, CPAP
Research, Coded, or Investigational Drug	ABL001, MK8669, BGB-A317
Vague or General Term (e.g., drug class)	ANTIDEPRESSANTS, PAINKILLER
Illegible or Nonsensical Data	QWDQFF, —
*Qualitatively Identified Themes from Manual Review (n=2,290)*	
Likely Natural Health Product (NHP) or Supplement	ADRENASENSE, SHILAJIT, MACA
Likely International or Non-Marketed Brand Name	ADRIBLASTINA, DOLIPRANE
Complex Multi-Ingredient Formula	BCAROTENEZNCUMINHVP, COGNITEX
Pipeline Miss / False Negative (e.g., misspelling)	ACIDE ACETYLSALYCILIQUE, BILASTINE

### Demonstrated impact on signal detection potential

Standardization significantly impacts data aggregation, which is critical for signal detection. For instance, mesalamine was reported under 15 distinct brand name variants (totaling 494 reports). None of these individual brand names generated a signal of disproportionate reporting (SDR) for asthenia: each had an IC metric with a 95% lower bound ≤ 0 (the highest being -0.01 for “ASACOL”, N=84). However, after aggregating all these reports under the generic name “mesalamine”, the consolidated data produced a significant SDR for asthenia. The IC_025 rose to 0.01 (IC point estimate 0.15) for N=446 aggregated reports. Similarly, the ROR became significant only after aggregation (ROR = 1.11, 95% CI lower bound = 1.01), whereas no individual brand variant had a significant ROR. In other words, standardization revealed a safety signal for asthenia associated with mesalamine consistent with known safety information [22–25]. This signal was previously obscured by name fragmentation.

Similarly, we observed an SDR for hydrochlorothiazide and erythema that became evident only after standardization. Ten different variants of hydrochlorothiazide (including entries like “APO-HYDROCHLOROTHIAZIDE”, “HCTZ”, “HydroDIURIL”, etc., representing a total of 335 reports) were associated with erythema, but none of these variants individually met the IC significance criterion (IC_025 ≤ 0 for all). One variant (“HCTZ”, N=57) was on the threshold (IC_025 = 0.0), and the most frequently reported variant (“HYDROCHLOROTHIAZIDE”, N=240) had IC_025 = -0.04. When all these reports were combined under the generic name “hydrochlorothiazide”, the aggregated analysis yielded a significant SDR for erythema: IC_025 increased to 0.03 (IC point estimate 0.19) for N=331 combined reports. The ROR likewise achieved statistical significance only after aggregation (ROR = 1.15, 95% CI lower bound = 1.03). This example further illustrates how drug name standardization can uncover safety signals (here, a dermatologic risk of hydrochlorothiazide) that were not detectable when the data were fragmented by name.

This emergent hydrochlorothiazide-erythema signal aligns with known risks of the drug [26,27]. The FDA-approved labeling for hydrochlorothiazide notes cutaneous adverse reactions, including photosensitivity and erythema multiforme, and the Canadian product monograph warns of severe skin reactions (such as toxic epidermal necrolysis and other erythema-related conditions). The fact that this signal was only detected after data aggregation demonstrates how our standardization pipeline can improve the sensitivity of signal detection by mitigating the dilution of signals caused by nomenclature variability.

## Discussion

In this study, we developed and validated a high-precision pipeline that successfully standardized over 94% of medication names in the Canada Vigilance database. This high coverage underscores the effectiveness of combining a multi-stage approach with external knowledge bases to handle the diverse and often noisy nomenclature found in real-world spontaneous reports. Our validated medication name standardization pipeline has significant practical implications for pharmacovigilance and health informatics, enhancing key pharmacovigilance activities and enabling broader interoperability of safety data.

### Pipeline performance and validation

A key strength of our study is the rigorous expert validation, which confirmed the pipeline’s exceptional reliability for its intended application. The very high precision (98.02%) and specificity (97.22%) are particularly important, confirming the pipeline’s suitability for pharmacovigilance applications where minimizing false positive signals is paramount to avoid unnecessary investigations and maintain trust in safety findings [3,28,29]. In other words, the pipeline rarely produced incorrect matches (false positives) and correctly identified true non-matches. The moderate recall (77.34%), resulting primarily from false negatives, reflects a deliberate design choice that prioritizes this high precision over exhaustive recall. Analysis of these false negatives revealed that they often involved complex synonymy (e.g., Aspirin vs. Acetylsalicylic acid) or uncaptured international variants. These cases highlight the challenging scenarios that may require nuanced domain knowledge beyond purely algorithmic solutions. Furthermore, the pipeline demonstrated qualitative reliability; for instance, it also appropriately left certain entries unmatched when they were not actual medications (e.g., medical devices like glucose meters) or when the input was unintelligible, preventing erroneous mappings of non-drug terms.

The analysis of the unmatched terms provides valuable insight into the pipeline’s performance and the limitations of standardizing real-world data. As shown in Table 4, a substantial portion of unmatchable entries were Natural Health Products (NHPs) and medical devices, categories for which RxNorm provides limited coverage. This finding has direct implications for pharmacovigilance; for example, the inability to systematically standardize NHPs could mask or dilute potential safety signals for this widely used class of products. This strongly supports our recommendation for future work to integrate Canadian-specific databases like Health Canada’s NHPID to close this coverage gap. Furthermore, the identification of a small number of “Pipeline Misses” (false negatives) is an important finding. It demonstrates that while the pipeline’s precision is very high, its recall is not perfect, and we recommend that researchers using this pipeline consider a secondary manual review for any specific unmatched terms of high interest to their research question. Finally, our analysis underscores that a notable fraction of raw “drug” data in SRS databases consists of terms that are not, in fact, standard pharmaceuticals, and handling these appropriately is a key challenge for data quality.

### Comparison with other standardization approaches

While other valuable standardization tools exist (for example, the DiAna dictionary tailored for FAERS which primarily targets RxNorm mapping [3], or WHODrug Koda which maps entries to WHODrug codes and inherent ATC classifications within VigiBase [15], our objective required a distinct approach. We aimed not only to standardize Canada Vigilance terms to RxNorm concepts (RxCUI) but also to consistently integrate both ATC classifications and detailed pharmacologic class information sourced directly via RxNav APIs within the RxNorm ecosystem. Furthermore, developing a custom, iterative pipeline allowed for specific tuning of preprocessing rules to address the unique characteristics observed in the Canadian data, including bilingual reporting patterns.

Our pipeline employs a hybrid approach, combining deterministic preprocessing rules with the sophisticated approximate matching capabilities of external, curated knowledge bases like RxNorm. This stands in contrast to end-to-end machine learning (ML) models, which represent an emerging alternative for name standardization.

The primary advantage of our methodology is its transparency and interpretability. Each step of the process is explicit, and the ‘special_cases.txt’ file provides a clear, auditable record of how specific known challenges are handled. By leveraging the vast, professionally maintained knowledge graph of RxNorm, our pipeline capitalizes on decades of curated terminological work without needing to re-learn these relationships from scratch.

An ML-based approach, such as a sequence-to-sequence neural network, could potentially offer greater flexibility in handling novel or unseen variations by learning latent patterns in language. However, this comes with significant trade-offs. Such models typically require a very large, high-quality, manually labeled dataset for training, which may not be readily available. Furthermore, they often function as “black boxes,” making it difficult to understand or correct why a specific incorrect mapping occurred. Our rule-augmented, API-driven approach was chosen as a pragmatic and robust solution that is both highly accurate and fully transparent, which is a critical consideration in the context of public health surveillance. Future work could explore hybrid systems that use ML to suggest mappings for terms that fail our current pipeline, combining the strengths of both approaches.

### Enhanced pharmacovigilance

Standardized drug nomenclature directly improves core pharmacovigilance tasks. By consolidating fragmented drug entries into unified concepts (as demonstrated in the Results section), the pipeline increases statistical power for signal detection, allowing for more accurate computation of drug-event frequencies and trends. This improved data clarity can lead to earlier detection of safety signals and more reliable assessment of adverse event patterns. As our case studies demonstrated, standardization can reveal safety signals, such as the known associations between mesalamine and asthenia or hydrochlorothiazide and erythema, that were previously obscured by name fragmentation. This ability to uncover otherwise latent signals demonstrates how our pipeline can improve the sensitivity of signal detection by mitigating the dilution effect of nomenclature variability. Furthermore, unifying drug entries under standard identifiers (RxCUI) and linking them to classifications (ATC) improves the accuracy of risk assessments and facilitates robust class-effect analyses. The high degree of enrichment achieved (>98% ingredient mapping, ∼74% ATC classification) significantly enhances the dataset’s utility for these downstream analyses. The automation also reduces the manual effort previously needed to clean and reconcile drug names, allowing analysts to focus on interpreting signals. Ultimately, by revealing signals that were previously obscured, our pipeline contributes to earlier detection of safety issues and enables more timely regulatory interventions.

### Broader applications and interoperability

Using international standards like RxNorm and ATC provides a foundation for improved data integration and interoperability. Standardized Canadian ADR data can be more readily compared with or merged into international pharmacovigilance databases, facilitating cross-database studies and validation of safety signals. This also future-proofs the data for integration into global surveillance networks that rely on common vocabularies. Key applications include:

- **Cross-Database Validation:** With standardized terminology, Canadian data can be more directly compared to or pooled with data from other sources (e.g., FAERS, VigiBase) using common identifiers (RxCUI, ATC) to validate signals across diverse populations and strengthen causality assessment.
- **Integration with Health Systems:** The methodology can be adapted for use in clinical information systems and Electronic Health Records (EHRs), standardizing local adverse event reports or enabling linkage of spontaneous ADR reports to clinical data (such as patients’ medication histories) for more comprehensive safety analyses.
- **Regulatory and Research Use:** Health authorities can leverage the standardized dataset for faster querying and retrieval of cases during safety investigations. Pharmaceutical companies can use this approach to harmonize diverse post-market data sources. Researchers benefit from integration into common data models like OHDSI/OMOP, enabling sophisticated pharmacoepidemiologic studies (e.g., temporal trends, comparative safety) that require consistent drug identifiers.

By transforming heterogeneous medication names into a consistent, enriched format, our pipeline improves data quality and interoperability. It strengthens signal detection within Canada, enhances the ability to share and interpret Canadian safety data in a global context, and provides a high-quality resource supporting evidence-based regulatory decisions and public health research.

### Generalizability and adaptability

While this pipeline was specifically developed and validated on the Canada Vigilance database, its core framework is designed to be generalizable. The fundamental challenges of medication name heterogeneity—including misspellings, brand/generic variations, and the presence of non-drug terms—are common across all spontaneous reporting systems, such as the FDA’s FAERS and the WHO’s VigiBase. The pipeline’s architecture, which relies on standard terminologies like RxNorm and modular components for preprocessing, matching, and enrichment, can serve as a robust template for standardizing other national ADR databases. However, direct application to another country’s data would require specific localization. The curated preprocessing files—unwanted_terms.txt and special_cases_canada.txt—are critical for the pipeline’s high performance and contain rules tailored to the Canadian context, such as handling of French-language terms and specific international brands common in Canada. To adapt the pipeline for another region, these lists would need to be reviewed and modified to incorporate that region’s own common drug names, local abbreviations, and linguistic conventions. This highlights a key principle: while the overall standardization framework is broadly applicable, achieving maximum accuracy in any new setting requires a degree of dataset-specific tuning and iterative refinement.

### Limitations and future directions

Despite the high performance of our pipeline, there are several limitations and areas for future improvement:

- **Reference Terminology Coverage:** The pipeline’s success is bound by the scope of reference terminologies (primarily RxNorm, supplemented by OHDSI vocabularies). If a medication (especially a newer or international product) is not represented in these sources, the pipeline cannot match it. This leaves a small subset of names unmatched, reflecting an inherent limitation of relying on existing vocabularies.
- **Ambiguity in Drug Names:** Addressing ambiguous or complex drug names remains challenging. In cases where an input name could correspond to multiple ingredients or products, the current pipeline may either pick one (risking an incorrect match) or return no match if uncertain. Additional strategies (such as probabilistic matching or incorporating context from the ADR reports) could help resolve such ambiguities.
- **Multilingual Support:** While we added basic support for French (the second official language in Canada), the pipeline’s multilingual capabilities are not exhaustive. It handles common French pharmaceutical terms and synonyms, but rarer or more complex multilingual cases might still be missed. Future enhancements could integrate more comprehensive multilingual dictionaries or translation components to improve coverage.
- **Validation Scope:** Our validation approach (a balanced sample of 200 cases reviewed by experts) was rigorous but limited in size. It may not capture all edge cases in the full dataset. Additionally, using two experts and requiring consensus means some borderline cases were excluded. In the future, expanding the validation set and involving more reviewers or a different adjudication process could further strengthen confidence in results.
- **Standardization Granularity:** The pipeline standardizes drug names primarily to the active ingredient level (or ingredient combination level). It does not distinguish between different products containing the same ingredient(s). While this is appropriate for most signal detection purposes, it could overlook formulation-specific issues (e.g., differences between an extended-release and an immediate-release product). Depending on the use case, finer-grained standardization or annotation might be needed.
- **External Dependency:** The pipeline relies on external APIs (RxNorm, OHDSI) for up-to-date information. This dependency means that network issues or API downtime can pause processing. Our caching mechanism mitigates this by allowing restarts without data loss, but a completely offline implementation would require maintaining local copies of these databases, which introduces challenges in data upkeep and infrastructure.
- **Rule-Based Approach:** Our solution is largely rule-based (augmented by external knowledge bases) rather than machine learning-based. A learning-based model (e.g., a neural network trained on known name mappings) could potentially handle some variations more flexibly, but it would require a substantial labeled dataset and would need careful evaluation to ensure it outperforms the current method.

In summary, these limitations point to opportunities for future work. Expanding reference vocabulary coverage, improving the handling of ambiguous and multilingual cases, exploring machine learning enhancements, and developing strategies for dealing with extremely complex reports (outliers) are all potential directions to further increase the pipeline’s robustness and utility.

## Reproducibility and transparency

Transparency and reproducibility were central to this research. The standardization pipeline (v9) was implemented in Python (v3.9) using standard libraries (e.g., Pandas, Requests) and relies on external APIs (RxNorm, OHDSI Athena, RxNav), with RxNorm access requiring a UMLS license. Deterministic processing and comprehensive caching ensure stability and consistent outputs. The primary output is a mapping file linking original drug names to standardized RxCUI and ATC codes. (Full implementation details are in the code repository documentation).

The complete source code, auxiliary files (curated term lists, special cases), and comprehensive documentation are publicly available on GitHub at: https://github.com/niazch/canada-vigilance-med-norm. This repository includes instructions for data acquisition, setup, and pipeline execution, enabling replication and adaptation. Supplementary materials, including the list of unmatched names, are also provided to foster scrutiny and community contribution, aligning with FAIR principles and promoting open science in pharmacovigilance [3].

The Canada Vigilance input data is publicly available and de-identified. Users should be aware of the inherent limitations of spontaneous reporting data (e.g., under-reporting), which standardization does not resolve, and that our standardized output, while highly accurate, is provided for research and may contain residual errors. All transformations are traceable for auditability.

## Conclusion

Medication name heterogeneity in spontaneous ADR databases represents a critical challenge for pharmacovigilance. We addressed this challenge by developing a high-precision pipeline that leverages advanced data standardization techniques to transform heterogeneous drug names into a harmonized resource, thereby enhancing signal detection and data quality for Canadian pharmacovigilance. Our iterative approach successfully standardized over 94% of reported drug names to RxNorm concepts, with the resulting data significantly enriched: ingredient identifiers were mapped for over 98% of matched terms, and ATC classifications were retrieved for approximately 74%. Expert validation confirmed the pipeline’s exceptional precision (98.02%) and specificity (97.22%).

The magnitude of the standardization problem was substantial: we identified 46,585 distinct drug name entries in the dataset, and 17,164 (∼36.85%) of these had no established ingredient mapping. Many of these unmapped names were combination products or non-standard terms that defied straightforward categorization. By standardizing both previously mapped and unmapped drug names, our pipeline bridges a major gap, transforming fragmented name variants into unified concepts suitable for analysis.

Our approach also tackled active ingredient heterogeneity. For example, a single substance like acetylsalicylic acid appeared under 149 different textual variations across 164 distinct ingredient identifiers in the raw data. The pipeline’s processing unifies such cases, ensuring that all reports referring to the same pharmacological entity are consolidated regardless of naming variation.

Expert validation provides strong evidence of the pipeline’s reliability. Domain specialists exhibited high agreement (κ = 0.8405) when assessing the pipeline’s output. This level of accuracy, particularly the high precision, is crucial in pharmacovigilance to avoid false signals that could misdirect safety efforts.

The significance of this work extends beyond technical data cleaning. By standardizing medication names, we directly enhance the sensitivity and specificity of safety signal detection, enabling regulators and researchers to identify potential drug risks more efficiently and accurately. It also facilitates the integration of Canadian pharmacovigilance data with international datasets, supporting broader safety assessments across different populations and healthcare systems. In essence, our approach strengthens evidence-based decision-making in drug safety surveillance.

## Supporting information

S1 Text Supplementary Methods and Analyses. This file contains detailed descriptions of the pipeline’s methodology, including additional figures (S1-S3 Figures) and tables (S1-S3 Tables) that are referenced within this text.
(PDF)

S2 Text Supplementary Results: Final Pipeline Validation. This file contains a detailed textual analysis of the expert validation results.
(PDF)
