# Supplementary material for: Enhancing adverse drug reaction data quality in Canada: A high-precision pipeline for medication name standardization and enrichment
Source: PLoS One. 2025 Sep 25;20(9):e0331940. doi: 10.1371/journal.pone.0331940 (PMC12463261; doi:10.1371/journal.pone.0331940)
Supplement: S1 Text — This file contains detailed descriptions of the pipeline’s methodology, including additional figures (S1-S3 Figures) and tables (S1-S3 Tables) that are referenced within this text. (PDF) [file pone.0331940.s001.pdf]

S1. Supplementary Methods

S1.1 Detailed Module Architecture

To provide deeper insight into the standardization pipeline's operation, we present detailed architectural diagrams for each primary module.

S1.1.1 Preprocessing Module

The preprocessing module (Fig S1) is the crucial first step in the standardization pipeline, designed to normalize the highly variable raw medication names extracted from the Canada Vigilance database and reduce noise that can significantly impede accurate downstream matching. This module implements a series of sequential text transformations:

1. **Case Normalization:** All input names are converted to lowercase to ensure case-insensitivity.

2. **Punctuation and Special Character Handling:** Standard punctuation (commas, parentheses) and special characters (e.g., +, ?, \, ;) are generally replaced with spaces. Specific logic handles ensuring spaces surround operators like '+' or '/' before their removal or replacement, aiding later tokenization. Non-alphanumeric characters (excluding hyphens, which may be part of some names) are typically removed. Specific patterns identified as noise (e.g., '(manufacturer unknown)') are also stripped out.

3. **Noise Term Removal via `unwanted_terms.txt`:** A comprehensive, curated list ('unwanted_terms.txt') containing several hundred terms is used to systematically remove noise from the name string. These terms, identified through iterative analysis of the Canada Vigilance data, are deemed non-essential for identifying the core drug concept. Table S1 provides a categorization and examples of these excluded terms.

Table S1. Categorization and Examples of Excluded Terms from `unwanted_terms.txt`.

Category	Description & Examples
**Pharmaceutical Forms**	Common dosage forms and preparations.<br>*Examples:* `tablet, capsule, injection, cream, comprime (French).`
**Dosage, Strength & Units**	Numeric values and common units of measurement.<br>*Examples:* `mg, ml, mcg, %.`
**Routes & Administration**	Terms describing how a drug is administered.<br>*Examples:* `oral, topical, intravenous, iv, im.`
**Release Mechanisms**	Terms indicating modified-release formulations.<br>*Examples:* `extended release, sr, er, retard, depot.`
**Manufacturer Names**	A large list of common pharmaceutical company names.<br>*Examples:* `pfizer, merck, sandoz, apotex, teva.`
**Filler & Conjunction Words**	Common words that add noise but no semantic value for matching.<br>*Examples:* `brand, generic, for, with, and, plus, unknown.`

4. **Abbreviation Expansion:** Common pharmaceutical abbreviations encountered in the data are expanded to their full form (e.g., 'hcl' to 'hydrochloride', 'hctz' to 'hydrochlorothiazide').

5. **Targeted Corrections via `special_cases.txt`:** This curated list was developed through an iterative, manual analysis of terms where the automated pipeline initially failed, produced an ambiguous match, or for which a direct local mapping was determined to be significantly more efficient. Each rule in this file represents a high-confidence mapping, often confirmed through domain expertise, designed to address several specific challenges:

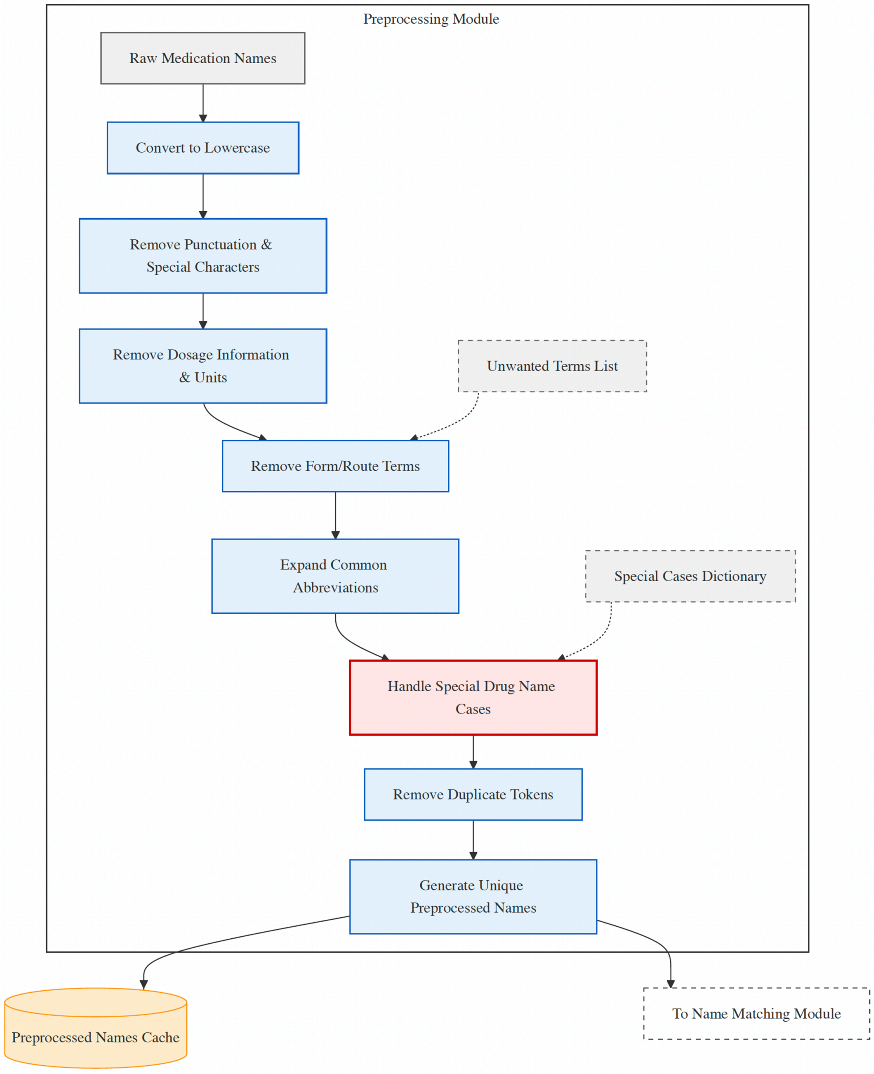

Figure S1. Preprocessing Module Architecture.

- **Resolving Ambiguity:** It provides a definitive mapping for terms known to cause ambiguous or incorrect matches with purely algorithmic approaches.
- **Mapping Complex and International Names:** It ensures accurate standardization for specific multi-ingredient products (e.g., mapping the supplement `metanx` to its components) or international brand names (e.g., `doliprane` to `Paracetamol`) that are not present in the core RxNorm terminology.
- **Handling Coded and High-Frequency Terms:** It guarantees the correct and efficient standardization of terms that are not discoverable via standard search, such as research codes (e.g., `lcz696` to `Sacubitril / Valsartan`), or other high-frequency non-standard phrases.

This targeted, knowledge-driven approach complements the generalized matching capabilities of the downstream modules. While this list is extensive, it represents a small fraction of the total unique names processed, and its modular design allows for future additions as new challenging terms are identified. The types of challenges addressed by this file are categorized and exemplified in Table S2.

6. **Tokenization and Deduplication:** The cleaned name string is tokenized (split into words, typically based on spaces and hyphens). Duplicate tokens are removed to prevent issues where repeated words

Table S2. Examples of Challenging Cases Addressed by the Curated `special_cases_canada.txt` File.

Challenge Type	Example of Targeted Correction Rule
**International Brand Not in RxNorm**	Maps a brand name common outside the U.S. that does not return a match from the RxNorm API. *Example:* `doliprane` → `Paracetamol`
**Complex Multi-Ingredient Product**	Provides a definitive mapping for a combination product, often a supplement, that lacks a pre-coordinated concept in RxNorm. *Example:* `metanx` → `l-methylfolate/...`
**Research or Coded Names**	Maps internal research codes or highly truncated names to their known active ingredients, which are not discoverable via standard search. *Examples:* `AIN457` → `Secukinumab`; `lcz696` → `Sacubitril / Valsartan`

might skew matching (e.g., "drug drug tablet" becomes "drug"). The unique tokens are then rejoined into the final preprocessed name string.

The output of this module is a normalized, cleaned string intended to represent the core pharmacological entity, stripped of much of the administrative, dosage, and manufacturer noise, and with specific known issues directly addressed, making it more amenable to successful matching against standard terminologies in the subsequent pipeline stage.

S1.1.2 Name Matching Strategy

The core standardization logic resides in the name matching module, which aims to assign the most appropriate RxNorm Concept Unique Identifier (RxCUI) to each unique preprocessed medication name derived from the initial stage (S1.1.1). This module employs a cascaded strategy, graphically represented in the main manuscript (Fig 3), prioritizing accuracy and efficiency by leveraging external terminology services and intermediate caching. The specific steps are as follows:

1. **Cache Check:** Before any external query, the system consults the persistent 'name_match_cache'. If a successful mapping (standardized name and RxCUI) for the given preprocessed name exists from a previous run or earlier in the current batch, this cached result is immediately retrieved and used, bypassing all subsequent API calls for that term. This significantly enhances performance on large datasets or during restarts.

2. **Primary RxNorm Query via `approximateTerm.json`:** For preprocessed names not found in the cache, the pipeline initiates a query against the RxNorm API using the 'approximateTerm.json' endpoint. This specific endpoint is intentionally utilized for its ability to perform sophisticated approximate matching, leveraging RxNorm's vast internal knowledge graph of synonyms, lexical variants, spelling corrections, abbreviations, and term relationships [1, 2]. We configure the API call to return only the single highest-ranked candidate match ('maxEntries=1'), entrusting the RxNorm algorithm to identify the most plausible concept based on its internal scoring mechanisms. The query effectively seeks the best fit, whether it represents an exact string match or a closely related term (e.g., corrected misspelling, brand-ingredient link).

3. **Match Acceptance:** If the 'approximateTerm.json' API call successfully returns a candidate concept (i.e., the query completes without error and identifies a relevant RxNorm term), this single top-ranked result is accepted as the standardized match. The associated RxCUI and standardized name are stored in the 'name_match_cache' for future use and passed forward to the enrichment stage (S1.1.4). No further local evaluation or confidence scoring is applied by our pipeline at this stage; acceptance relies solely on the RxNorm API providing a top candidate.

4. **Handling Primary Query Failure & Fallback Initiation:** If the primary RxNorm query (Step 2) fails to return any candidate match—which can occur if the term is not recognized by RxNorm (e.g., a

non-drug term or an international product not covered) or if a transient API connectivity issue occurs (which is logged separately)—the pipeline proceeds to a fallback mechanism.

5. **Fallback OHDSI Athena API Query:** The preprocessed name that failed the primary RxNorm query is submitted to the OHDSI Athena API. This API provides access to a broader range of international medical vocabularies, including the RxNorm Extension, which contains concepts not present in the core RxNorm dataset. The query specifically searches for concepts within relevant domains (e.g., 'Drug') potentially matching the input term, aiming to identify international brand names or alternative representations. Our script's ‘fetch_notmatched‘ function handles this query, incorporating timeout mechanisms to manage potential API sluggishness.

6. **Processing OHDSI Result:** The JSON response from the Athena API is parsed by the ‘extract_international_names‘ function. This function applies specific logic to identify the most likely relevant concept name from the results, prioritizing 'Standard' concepts in the 'Drug' domain or names explicitly linked to the input term via bracketed annotations. If a plausible alternative name is extracted, it undergoes the same preprocessing steps (S1.1.1) as the original names to normalize it.

7. **Secondary RxNorm Query:** The normalized alternative name identified via OHDSI is then submitted back to the RxNorm ‘approximateTerm.json‘ API endpoint (‘maxEntries=1‘), representing a second attempt to obtain a core RxNorm RxCUI. This step aims to anchor even internationally identified terms within the primary RxNorm framework if possible.

8. **Final Outcome:**
   - If the secondary RxNorm query (Step 7) returns a successful match, that RxCUI and standardized name are accepted and cached.
   - If the OHDSI query (Step 5) fails to find a plausible alternative name, or if the secondary RxNorm query (Step 7) also fails to return a match, the original preprocessed name is considered unmatchable by the pipeline and is marked accordingly.

This comprehensive multi-step process, combining direct reliance on the RxNorm API's matching capabilities with a targeted OHDSI fallback, maximizes the opportunity to standardize terms while maintaining RxNorm as the authoritative target vocabulary.

S1.1.3 Multi-tiered Caching System

Performance optimization is achieved through a sophisticated multi-tiered caching system (Fig S2). The pipeline maintains separate JSON cache files for each processing stage: preprocessed names, name-to-RxCUI mappings (effectively caching RxNorm match results), OHDSI query results (for fallback lookups), and medication metadata (enrichment results). This architecture significantly reduces redundant external API calls, optimizing processing time. Equally importantly, it provides resilience against network connectivity issues or other interruptions; by loading cached data upon startup, the pipeline avoids reprocessing already completed names. The caching architecture also enables incremental updates, when new medication names are added to the dataset, only these new entries require full processing through the API-dependent stages, with existing mappings retrieved rapidly from local cache files.

S1.1.4 External Data Enrichment

The external data enrichment module (Fig 4 in the main text) augments successfully matched entries (i.e., those assigned an RxCUI via the matching process) with comprehensive pharmacological information derived solely from RxNav APIs.

For each matched medication RxCUI, the module executes a sequence of calls to retrieve:

1. **Related Ingredients:** Queries RxNav (related.json?tty=IN) to find the associated active ingredient(s), capturing their names and RxCUIs. This ingredient identification step proved highly effective in our final analysis, succeeding for over 98% (98.66%) of entries matched to an RxCUI in the Canada Vigilance dataset.

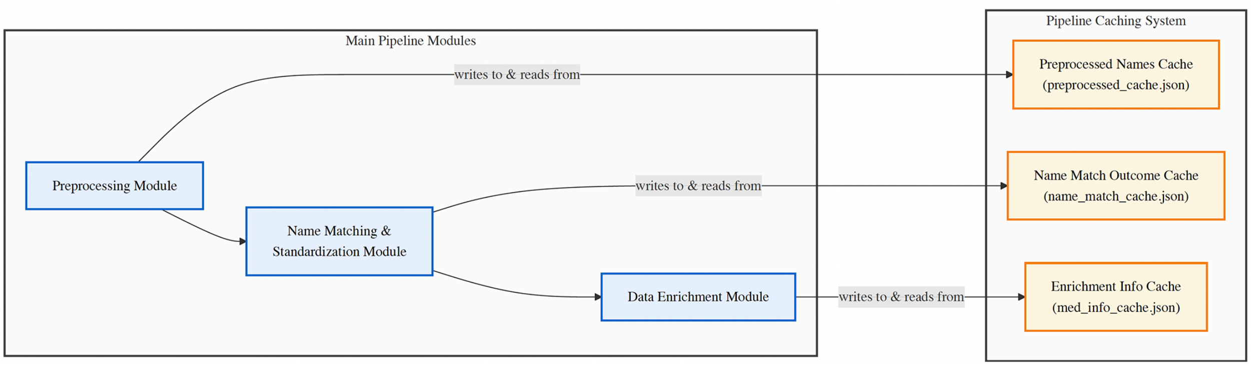

Figure S2. Multi-tiered Caching System.

2. **Generic Name Representation:** Fetches a standardized generic name using RxNav functions. (Note: The script function get_generic_name_from_openfda primarily queries RxNav endpoints).

3. **Drug Class:** Queries the RxClass API within RxNav (class/byRxcui.json) using sources like FDASPL to identify associated drug classes (e.g., Established Pharmacologic Class).

4. **ATC Classification:** Queries the RxClass API within RxNav (class/byRxcui.json), typically using the ingredient RxCUI and the ATC relaSource, to retrieve the relevant ATC code (usually level 4) and its descriptive name. In our analysis, this step successfully populated ATC-4 codes for approximately 74% (73.81%) of the matched entries, providing valuable therapeutic context for a substantial majority.

The system incorporates robust error handling, with timeout-based retries for API calls and fallback mechanisms that preserve partial information when complete enrichment isn't possible. This enrichment process transforms simple name standardization into a rich, context-aware dataset suitable for sophisticated pharmacoepidemiological analyses, characterized by near-complete ingredient mapping and broad, though not universal, ATC classification coverage for matched terms.

S1.1.5 Output Processing

The final output processing module, illustrated in Fig S3, consolidates the standardized results into structured formats for analysis and distribution. Matched names with their complete enrichment data are mapped back to the original names and assembled into a primary dataframe. Unmatched names are collected separately for potential further analysis or manual review. The module generates summary statistics on match rates and standardization quality, outputting both the standardized dataset (in CSV format) and comprehensive logging information for transparency and validation.

S1.2 Iterative Methodological Refinement

The development of our medication name standardization framework has been characterized by iterative methodological enhancements, each informed by systematic evaluations of performance, coverage, and accuracy. Initially conceived as a basic prototype, the earliest version relied solely on simple text normalization, lowercasing input strings and removing punctuation, and then attempted an approximate match to RxNorm. While this proved the feasibility of automated standardization, the high proportion of unmatched or ambiguously matched names quickly revealed the complexities posed by real-world medication data.

Recognizing these limitations, subsequent iterations refined the preprocessing phase. Advanced tokenization strategies were introduced to dynamically handle special characters (e.g., ”+,” ”?,” parentheses) and to segment compound names. We implemented systematic expansions for common abbreviations, for example, mapping ”hcl” to ”hydrochloride” and ”sr” to ”sustained release.” This step also included targeted replacement rules for special cases known to confound the baseline system. These enhancements improved coverage and reduced preprocessing errors, providing a more robust foundation for downstream matching.

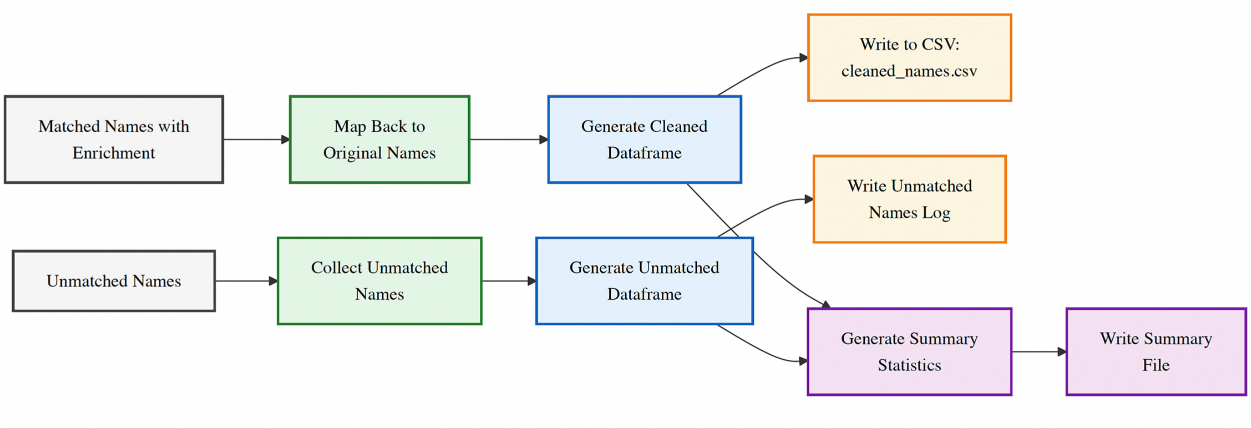

Figure S3. Output Processing Module.

As we progressed, fuzzy matching capabilities were integrated to handle names that remained unmatched after exact matching. Initial explorations (corresponding to improvements seen around v3) utilized local algorithms based on string similarity metrics (e.g., Levenshtein distance via Python's difflib or other libraries). While helpful, these local methods were later superseded by leveraging RxNorm's more sophisticated approximateTerm.json endpoint, which incorporates deeper terminology knowledge. This API-based approach proved especially useful for spelling variants, typographical errors, or alternate international spellings (e.g., "cyclosporine" vs. "ciclosporin"), significantly increasing recall without sacrificing precision.

To further expand coverage of non-U.S. or combination products, we incorporated additional data sources beyond RxNorm. The OHDSI vocabulary, including RxNorm Extension concepts, was queried for intractable cases. This approach enabled the identification and mapping of international brand names, as well as multi-ingredient products reported under a single brand. When names were clearly composed of multiple active ingredients joined by a slash or other delimiters, we split them accordingly and attempted a separate match for each component.

The expanding range of data sources inevitably increased computational overhead, prompting substantial performance optimizations. We introduced a multi-tiered caching mechanism to reduce redundant external queries, storing intermediate results such as preprocessed names, successful matches, and enriched medication attributes. Batch processing further optimized disk I/O and stabilized performance under high data volumes. These efficiency gains proved essential for maintaining practical processing times when iterating on the algorithm or re-running the pipeline on large datasets.

In parallel, we enhanced the output to include additional metadata, such as generic ingredient names, Anatomical Therapeutic Chemical (ATC) codes, and relevant drug-class information, to support pharmacoepidemiological analyses. Error-handling routines were also reinforced: if the RxNorm API timed out or provided inconsistent data, the system would either retry the query or log the failure for subsequent reprocessing. Through these refinements, the pipeline evolved into a resilient tool capable of both accurately mapping medication names to standardized terminologies and enriching those matches with clinically relevant information.

To accommodate multilingual considerations, a later iteration introduced rules for recognizing and removing common French descriptors (e.g., "comprimé," "injectable") frequently appearing in Canadian datasets. We also included French synonyms for certain active ingredients to improve the standardization of bilingual data. By the final version, fewer than 5% of medication names remained unmatched; these typically included obscure nutraceuticals, products misclassified as medications, or highly novel compounds absent from our reference dictionaries.

Throughout this iterative process, quantitative assessments were integral to evaluating and guiding each update. At every stage, we monitored metrics such as match rate, processing time, and error incidence. This empirical feedback loop identified new classes of problematic names, prompting targeted additions to dictionaries or adjustments to fuzzy matching thresholds, and confirmed when refinements improved both coverage and precision.

Over the course of nine iterations (v1 to v9), each key change in the pipeline yielded notable gains in coverage. After v1's basic RxNorm lookup (58.7% coverage), v2 introduced simple text cleanup (term removal), raising the match rate to 65.4%. v3 added fuzzy matching, substantially boosting coverage to 75.1%. In v4, combining OHDSI-based synonym integration improved coverage further to 82.3%. v5 primarily addressed performance via caching, with a modest gain to 82.5%. Adding ATC enrichment in v6 had no direct effect on coverage. By v7, addressing edge cases raised coverage to 85.0%. v8's multilingual support for French terms elevated it to 87.2%, and finally, v9 applied targeted manual corrections to achieve the highest coverage of 94.5%

The final version (v9) represented a substantial advancement in standardization capability, error resilience, and processing efficiency. While v8 had introduced comprehensive French term handling, v9 implemented a sophisticated error management framework capable of tracking and recovering from connectivity issues with external APIs, a critical feature for processing large-scale datasets. The pipeline's architecture was restructured to introduce a modular caching system with separate persistent storage for each processing stage: preprocessed name transformations, RxNorm API responses, standardized name mappings, and enriched medication information. This modular approach enabled selective reprocessing of specific pipeline components without redundant API calls, significantly enhancing both performance and flexibility. Another key innovation was the implementation of a cascade resolution strategy for challenging medication names; when standard RxNorm matching failed, the system automatically queried the OHDSI vocabulary for international variants, reprocessing these alternatives through the standardization pipeline. This international fallback mechanism substantially improved coverage for non-US drug products common in Canadian reporting. V9 also introduced comprehensive performance analytics that automatically quantified match rates, API timeout frequencies, and standardization failures, enabling data-driven refinement of problematic name patterns. The implementation of configurable batch processing with intelligent memory management further optimized performance for large datasets. These enhancements collectively increased the match rate from 87.2% (v8) to 94.5% (v9), representing thousands of additional successfully standardized medication names in the Canada Vigilance dataset.

This staged refinement process, evaluated quantitatively at each step (as shown in Table 1 of the main manuscript), not only demonstrates the path to the final high-performing pipeline but also offers practical lessons on the effectiveness of various techniques for tackling nomenclature heterogeneity in real-world pharmacovigilance data.

S1.3 Algorithmic Justifications

Our algorithmic choices were guided by the fundamental requirements for processing heterogeneous medication data: maximizing coverage while maintaining accuracy, achieving computational efficiency, and ensuring clinical validity. The rationale for key methodological decisions is presented below:

- **Fuzzy String Matching via RxNorm API:** A significant challenge in ADR data is the prevalence of spelling variations, typographical errors, and international naming differences that cause exact matching to fail. While developing custom synonym dictionaries and local fuzzy matching algorithms (e.g., based on Levenshtein distance) is one approach, it is often labor-intensive, requires continuous maintenance, and may lack the nuanced understanding embedded in clinical terminologies. Therefore, we chose to leverage the RxNorm API's approximateTerm.json endpoint for fuzzy matching. This approach offers several advantages: it utilizes RxNorm's extensive internal knowledge base, including synonyms, known variants, and term relationships, to provide contextually relevant matches ranked by confidence. By relying on this established, externally maintained resource, we benefit from its sophisticated matching logic and ongoing updates without the overhead of developing and maintaining a comparable local system. This decision prioritized leveraging existing, high-quality terminology resources to handle approximate matching effectively and efficiently [1].

- **External API Integration vs. Local Dictionaries:** The decision to leverage online APIs (RxNorm, OHDSI) rather than static local dictionaries was motivated by several factors: (1) ensuring access to continuously updated terminology as new drugs enter the market, (2) benefiting from the sophisticated relationship modeling these systems provide (e.g., ingredient-to-brand mappings), and (3) reducing the maintenance overhead associated with local vocabulary management. The integration with these

established knowledge bases significantly enhanced both coverage and reliability, enabling the system to leverage expert-curated terminological relationships. To mitigate potential network dependencies, our comprehensive caching system ensures operational stability even when external services experience temporary unavailability.

- **Noise Reduction through Preprocessing:** Our preprocessing rules were systematically developed based on empirical analysis of their effect on match quality. The removal of dosage forms, strength specifications, and administrative qualifiers was justified by the observation that such elements frequently obstruct successful matching while rarely contributing to core drug identity. Each preprocessing rule underwent validation to ensure it improved overall match rates without introducing inappropriate term conflation. This approach substantially reduced the dimensionality of the matching problem by focusing on core medication concepts rather than their administrative or dosage specifications.

These algorithm selections represent deliberate tradeoffs between competing requirements, collectively forming a system optimized for handling the complexities of real-world pharmacovigilance data. By combining deterministic approaches (for high-confidence cases) with probabilistic methods (for ambiguous entries), the pipeline achieves both precision and recall while maintaining computational efficiency.

S1.4 Integration of External Knowledge Sources

A major factor in the success of our standardization pipeline is the integration of external knowledge bases, which provide authoritative information beyond what could be inferred from the raw data alone. We specifically integrated the following sources in defined roles:

- **RxNorm / RxNav (NLM):** The RxNorm terminology, accessed via RxNav APIs, served as the backbone for both matching and enrichment. The approximateTerm.json API endpoint was the primary tool for mapping potentially misspelled or variant raw names to standardized concepts and obtaining a unique RxNorm Concept Unique Identifier (RxCUI). This structured approach ensures precision, which is crucial for distinguishing similar-sounding or similarly-spelled medications. For example, "Dilantin" (an anticonvulsant brand) and "Diltiazem" (a calcium channel blocker) share some letters; a naive string match could confuse them, but RxNorm maps "Dilantin" definitively to phenytoin's RxCUI and "Diltiazem" to its own distinct RxCUI, preventing conflation. Once an RxCUI was identified, further RxNav API calls were used exclusively for enrichment, retrieving its ingredient(s), associated drug classes (via RxClass), and ATC classification (also via RxClass, typically linked to the ingredient). RxNorm's structure of linking multiple vocabularies means finding an RxCUI provides a robust anchor for reliable data integration.

- **OHDSI Athena API:** The OHDSI vocabulary, accessed via the Athena API, played a distinct and specific role as a fallback lookup source. It was not used for primary enrichment (like obtaining ATC codes directly from its concepts). Instead, when the initial RxNorm API query failed to match a term, we queried the OHDSI Athena API (which includes vocabularies like RxNorm Extension) to search specifically for potential international or alternative names associated with the original term. If a plausible alternative was found, that name was then re-processed through the RxNorm approximateTerm.json API to attempt a match and obtain an RxCUI. This OHDSI-based fallback step was crucial for improving coverage, particularly for non-US drug products common in Canadian reporting, but the final standardization and enrichment data points relied on the concepts and relationships within the RxNorm/RxNav ecosystem.

- **ATC Classification Source:** It is important to reiterate that while the OHDSI ecosystem heavily utilizes ATC, in our pipeline, the ATC codes and names associated with a matched RxCUI were retrieved directly via the RxNav API's RxClass functionality. This ensures the ATC classification is directly and authoritatively linked to the standardized RxNorm concept identified by our pipeline.

By combining these sources in a defined sequence, RxNav for primary matching and all enrichment, with OHDSI providing a targeted fallback lookup, our pipeline leverages the strengths of each resource appropriately. This integration enhances both coverage (by finding international names via OHDSI) and

reliability (by standardizing to recognized RxNorm identifiers and enriching via authoritative RxNav APIs). Furthermore, the use of standard identifiers like RxCUI and ATC lends credibility to the standardization process, increasing trust in the cleaned data within the pharmacoepidemiology community.

S1.5 Experimental Setup

To evaluate the effectiveness of our medication name standardization methodology, we conducted experiments on a comprehensive dataset of drug names extracted from the Canada Vigilance ADR database. The dataset consisted of all unique medication names appearing in reports over a multi-year period (exact range: 1965-2024 as per the latest data extract). This resulted in approximately 46,586 distinct drug name entries to be standardized (after initial parsing of the raw data files). These names served as the input to our pipeline. We split the evaluation into two parts: (1) retrospective analysis of iterative improvements using historical pipeline versions, and (2) final performance assessment of the completed pipeline on the full dataset.

For retrospective analysis, we replayed earlier pipeline versions on a subset of the data (or the whole data when feasible) to quantify how each iteration improved the outcomes. Key performance metrics included:

- *Match Rate:* For quantitative evaluation, the primary performance metric, Match Rate, was defined as the percentage of unique original drug names in the input dataset that were successfully mapped to a non-null RxNorm Concept Unique Identifier (RxCUI) by the pipeline.
- *Unmatched Count:* the number (and % proportion) of names that remained without a match.
- *Error Rate:* Though harder to quantify without a gold standard, we estimated errors by spot-checking a sample of matched outputs to see if any were incorrect mappings (false positives). Additionally, ambiguous mappings (where a name could map to multiple drugs) were flagged and counted.

We used these metrics to compare versions v1 through v9. Each version was run in a controlled environment (same hardware, consistent external API usage) to ensure differences were due to algorithm changes rather than external factors. For statistical analysis, we treated the final pipeline's results as the primary outcome and earlier versions as baseline comparators. We used McNemar's test for changes in match status of individual items (e.g., to test if the improvement from version to version in matched vs unmatched distribution was significant) and simple descriptive statistics for overall percentages. Since our dataset is basically the entire population of names, formal hypothesis testing is less critical than demonstrating clear improvement trends, but we included it for completeness.

Additionally, we conducted a qualitative evaluation: we manually reviewed a random sample of 100 names from the final output (50 that were matched by the pipeline and 50 that remained unmatched) to assess correctness and to understand the nature of remaining challenges. For matched names, we checked whether the assigned standardized name truly corresponded to the original (by consulting drug references). For unmatched, we tried to determine why (e.g., obscure product, incomplete name, etc.) to categorize the gaps.

S1.6 Validation Methodology

We constructed a validation set of 200 medication name pairs derived from the pipeline's output, comparing original names to their algorithm-assigned standardized names. While the operational dataset is highly skewed towards successful matches (over 94%), a balanced validation set was intentionally constructed, comprising 101 pairs where our algorithm identified similarity ("YES") and 99 pairs where it identified dissimilarity ("NO"). This balanced approach ensures a sufficient number of both potential matches and non-matches, as determined by the algorithm, are included. It enables a robust evaluation of the pipeline's performance characteristics across different scenarios, particularly its ability to correctly identify true non-matches (specificity) and to characterize failure modes (false negatives among the "NO" decisions). Reliably assessing performance on the minority class (algorithm identifying dissimilarity) would be statistically challenging using a purely prevalence-based sample drawn from the heavily skewed operational output.

The total sample size of 200 was selected as a pragmatic balance between the intensive manual labor required for dual-expert review and the need to achieve stable estimates for our primary accuracy metrics.

As the primary goal of this validation was to *estimate* the pipeline's performance metrics (e.g., precision, specificity) rather than to test a formal statistical hypothesis of effect, a formal *a priori* sample size calculation based on statistical power was not performed. This approach aligns with modern standards for diagnostic accuracy and model validation studies, where the objective is quantification and precision rather than null-hypothesis testing [13, 16, 17]. The statistical precision achieved with our sample size is, therefore, transparently reported using 95% confidence intervals for all key performance metrics, which is the recommended practice for communicating the certainty of performance estimates in such studies [14, 15].

Two independent domain experts reviewed each medication name pair, determining whether the original and standardized names represented the same medication. We defined expert consensus as cases where both experts agreed on a "YES" determination, taking a conservative approach to establishing the reference standard. The experts were blinded to the algorithm's decisions to prevent bias.

S1.7 Impact on Report Complexity and Signal Detection Methodology

Beyond quantitative performance metrics and expert validation, the true measure of the standardization pipeline lies in its ability to enhance pharmacovigilance practice. We now present case examples demonstrating how consolidating fragmented drug nomenclature through our validated methodology led to the emergence of potentially significant safety signals.

Our analysis identified instances where drug name standardization led to the emergence of previously undetected signals of disproportionate reporting (SDRs). A notable example involves the association between mesalamine and asthenia. In the analysis using specific brand names, none of the 15 distinct brand name variations associated with mesalamine (e.g., ASACOL, MEZAVANT, PENTASA, SALOFALK) generated an SDR for asthenia when assessed individually using the Information Component (IC) metric against the database background; all exhibited a lower bound of the 95% credibility interval (IC025) less than or equal to the significance threshold of 0. The highest IC025 observed among these individual brands was -0.01 (for "ASACOL", N=84). Collectively, these non-signaling brand-event reports represented 494 observations. However, upon aggregating these reports under the standardized generic term "mesalamine", the consolidated analysis yielded a statistically significant SDR based on the IC metric (N=446, IC025 = 0.01, IC point estimate = 0.15). This finding was further supported by the Reporting Odds Ratio (ROR) analysis; the standardized generic analysis also yielded a significant ROR (ROR = 1.11, 95% CI Lower Bound = 1.01), whereas the ROR had not met the significance threshold (Lower Bound > 1 and N ≥ 3) for the individual brand name variations. This demonstrates how standardization revealed a signal for asthenia, supported by both Bayesian and frequentist metrics, that was obscured when analyzing fragmented brand-name data. This emergent signal for mesalamine and asthenia, detected only after drug name standardization, aligns with known safety information for the drug. Specifically, asthenia and/or fatigue are listed as adverse reactions in the FDA-approved prescribing information for multiple mesalamine formulations, including LIALDA® (where asthenia is noted as "common" with 1-10% frequency) and PENTASA® [3, 4]. Furthermore, independent analysis of the FDA Adverse Event Reporting System (FAERS) and others has also identified fatigue and asthenia, associated with mesalamine [5, 6]. The detection of this known association only following the aggregation of data under the standardized generic name underscores the potential for name fragmentation to mask signals in pharmacovigilance analyses.

Similarly, an emergent SDR was detected for hydrochlorothiazide and erythema following drug name standardization. The analysis identified 10 different brand names or combinations involving hydrochlorothiazide (e.g., APO-HYDROCHLOROTHIAZIDE, HCTZ, HYDRODIURIL) reported in conjunction with erythema. None of these individual entries met the criteria for an SDR based on the IC metric (IC025 ≤ 0). One specific entry ("HCTZ", N=57) reached the threshold boundary (IC025 = 0.0), while the most frequently reported non-signaling entry ("HYDROCHLOROTHIAZIDE", N=240) had an IC025 of -0.04. These 10 non-signaling brand-level entries accounted for a total of 335 observations. When these reports were standardized under the generic name "hydrochlorothiazide", the resulting analysis produced a significant SDR based on the IC metric (N=331, IC025 = 0.03, IC point estimate = 0.19). This emergent signal was corroborated by the ROR metric, which also achieved statistical significance only following standardization (ROR = 1.15, 95% CI Lower Bound = 1.03). This case further illustrates the potential for drug name standardization to uncover safety signals, like erythema with hydrochlorothiazide, by consolidating reporting data. This emergent signal for hydrochlorothiazide and erythema, identified following drug name standardization, corresponds

with well-established dermatologic risks associated with this medication. Hydrochlorothiazide's potential to cause cutaneous reactions is recognized in its FDA-approved prescribing information, which lists both photosensitivity and erythema multiforme as potential adverse reactions [7]. Additionally, the Canadian Product Monograph for hydrochlorothiazide acknowledges the risk of severe cutaneous adverse reactions, including toxic epidermal necrolysis (TEN) and other erythema-related manifestations [8]. The recovery of this signal through data aggregation highlights how name standardization can improve the detection sensitivity for known drug-event associations, potentially diluted by reporting variability.

These emergent signals were identified using a report-based unit of analysis for calculating the Information Component (IC) and Reporting Odds Ratio (ROR). This approach was selected due to the specific characteristics of the Canada Vigilance data, notably the presence of reports with extreme complexity.

To quantitatively assess the impact of drug name variability on apparent report complexity within the Canada Vigilance database, we calculated potential drug-event pair counts per report using both the raw reported drug entries and standardized generic names derived from our mapping process. Table S3 presents these comparative metrics for the 20 reports exhibiting the highest complexity based on raw pair counts. A striking reduction in complexity is observed following standardization. For instance, the top-ranked report (Report ID: 903921327) contained 1,615 raw drug mentions corresponding to only 68 distinct generic drug concepts, resulting in a decrease in calculated drug-event pairs from 134,045 to 5,644 (a reduction of approximately 96%). Similar substantial reductions in both the effective drug count (comparing raw_drug_count to distinct_generic_drug_count) and the resulting pair counts (pairs_per_report_raw vs. pairs_per_report_standardized) are evident across all top 20 reports. This analysis highlights that a significant component of the high apparent complexity in certain reports stems from multiple synonymous reported terms mapping to a smaller set of unique pharmacological entities. These findings underscore the critical importance of robust drug name standardization for accurate interpretation of pharmacovigilance data and for potentially mitigating issues like inflated background rates in downstream analyses such as disproportionality signal detection.

Table S3. Reduction in Apparent Report Complexity Metrics Following Standardization for Top 20 Most Complex Reports (by Raw Pair Count).

**Report ID**	**Raw Drug Count**	**Distinct Generic Drug Count**	**Reaction Count**	**Raw Drug-Event Pairs**	**Standardized Drug-Event Pairs**
903921327	1615	68	83	134045	5644
994886	1058	122	64	67712	7808
905857350	417	61	152	63384	9272
1043994	373	82	166	61918	13612
1107540	365	86	161	58765	13846
904193203	321	45	178	57138	8010
900803504	366	60	148	54168	8880
1008281	436	75	123	53628	9225
906825093	405	72	116	46980	8352
900463532	458	60	100	45800	6000
1028218	374	76	119	44506	9044
907125549	422	46	100	42200	4600
906867001	418	55	100	41800	5500
907452627	463	56	90	41670	5040
1032323	891	120	45	40095	5400
907351864	280	74	140	39200	10360
906327899	312	73	125	39000	9125
907314732	356	64	109	38804	6976
907300609	256	70	151	38656	10570
1029948	329	71	117	38493	8307

Our analysis revealed substantial heterogeneity not only in nomenclature but also in report complexity within the Canada Vigilance database. We identified reports containing exceptionally high numbers of co-reported drugs and reactions, leading to calculated raw drug-event pair counts exceeding 130,000 for a single report. While our standardization process significantly reduces the drug count component of this

complexity by consolidating synonymous terms, the presence of such high-volume outlier reports represents a notable characteristic of this data source. This poses potential methodological challenges for certain downstream analyses, particularly standard pair-based disproportionality methods commonly used in signal detection [9, 10]. The disproportionate contribution of these outlier reports to overall pair counts may inflate background statistics, potentially masking weaker signals arising from more typical reports or skewing results, an effect specifically highlighted in pharmacovigilance literature concerning pair-based analyses [9]. Recognizing this potential bias within the dataset during our analysis, the signal detection examples presented in the Results section of the main manuscript employed a report-based unit of analysis (specifically using the Information Component, IC) as a pragmatic approach suitable for this specific context. Report-based methods are considered more robust to such variations in report complexity as they treat each report as a single observational unit [9, 10]. Furthermore, Bayesian report-based methods like the IC tend to exhibit greater stability and lower false-positive rates compared to frequentist pair-based alternatives, particularly in heterogeneous datasets [11, 12].

Consequently, a limitation when using this dataset for standard pair-based analyses is the need to account for these extreme outliers. Future research directions should therefore include a dedicated investigation into the nature and data quality of these high-complexity reports. Furthermore, comparative studies evaluating the robustness and utility of different analytical units (e.g., report-level versus pair-level disproportionality analysis) [12] and the application of specific outlier detection or handling techniques [9] within the context of the Canada Vigilance database are warranted to determine the most reliable approaches for signal detection in this specific pharmacovigilance resource.

References

[1] Waters R, Malecki S, Lail S, Mak D, Saha S, Jung HY, et al. Automated identification of unstandardized medication data: a scalable and flexible data standardization pipeline using RxNorm on GEMINI multicenter hospital data. JAMIA open. 2023;6(3):ooad062.

[2] U S National Library of Medicine. Approximate matching in the RxNorm API; 2015. Available from: https://lhncbc.nlm.nih.gov/RxNav/news/RxNormApproxMatch.html.

[3] U S Food and Drug Administration. Approval Package for Lialda (mesalamine); 2011. https://www.accessdata.fda.gov/drugsatfda_docs/nda/2011/022000Orig1s005.pdf.

[4] U S Food and Drug Administration. PENTASA® (mesalamine) extended-release capsules prescribing information; 2021. https://www.accessdata.fda.gov/drugsatfda_docs/label/2021/020049s036lbl.pdf. Available from: https://www.accessdata.fda.gov/drugsatfda_docs/label/2021/020049s036lbl.pdf.

[5] Sehgal P, Colombel JF, Aboubakr A, Narula N. Systematic review: safety of mesalazine in ulcerative colitis. Alimentary pharmacology & therapeutics. 2018;47(12):1597–1609.

[6] Liu M, Gu L, Zhang Y, Zhou H, Wang Y, Xu ZX. A real-world disproportionality analysis of mesalazine data mining of the public version of FDA adverse event reporting system. Frontiers in Pharmacology. 2024;15:1290975.

[7] Hydrochlorothiazide Side Effects; 2020. Available from: https://www.drugs.com/sfx/hydrochlorothiazide-side-effects.html.

[8] Sanis Health Inc . Hydrochlorothiazide Product Monograph. Sanis Health Inc.; 2021. Available from: https://dis-prod.assetful.loblaw.ca/content/dam/loblaw-companies-limited/creative-assets/sanis/2021/leaflets/HYDROCHLOROTHIAZIDE-leaflet.pdf.

[9] Juhlin K, Ye X, Star K, Norén GN. Outlier removal to uncover patterns in adverse drug reaction surveillance–a simple unmasking strategy. Pharmacoepidemiology and drug safety. 2013;22(10):1119–1129.

[10] Uppsala Monitoring Centre. Measures of Disproportionate Reporting. WHO Collaborating Centre for International Drug Monitoring, The Uppsala Monitoring Centre; 2016. Available from: https://who-umc.org/media/164041/measures-of-disproportionate-reporting_2016.pdf.

[11] Caster O, Aoki Y, Gattepaille LM, Grundmark B. Disproportionality analysis for pharmacovigilance signal detection in small databases or subsets: recommendations for limiting false-positive associations. Drug Safety. 2020;43:479–487.

[12] Park G, Jung H, Heo SJ, Jung I. Comparison of data mining methods for the signal detection of adverse drug events with a hierarchical structure in postmarketing surveillance. Life. 2020;10(8):138.

[13] Lakens D. Sample size justification. Collabra: psychology. 2022;8(1):33267.

[14] Bachmann LM, Puhan MA, Ter Riet G, Bossuyt PM. Sample sizes of studies on diagnostic accuracy: literature survey. Bmj. 2006;332(7550):1127–1129.

[15] Akoglu H. User's guide to sample size estimation in diagnostic accuracy studies. Turkish Journal of Emergency Medicine. 2022;22(4):177–185.

[16] Riley RD, Collins GS, Ensor J, Archer L, Booth S, Mozumder SI, et al. Minimum sample size calculations for external validation of a clinical prediction model with a time-to-event outcome. Statistics in medicine. 2022;41(7):1280–1295.

[17] Rothman KJ, Greenland S. Planning study size based on precision rather than power. Epidemiology. 2018;29(5):599–603.
