# Supplementary material for: Enhancing adverse drug reaction data quality in Canada: A high-precision pipeline for medication name standardization and enrichment
Source: PLoS One. 2025 Sep 25;20(9):e0331940. doi: 10.1371/journal.pone.0331940 (PMC12463261; doi:10.1371/journal.pone.0331940)
Supplement: S2 Text — This file contains a detailed textual analysis of the expert validation results. (PDF) [file pone.0331940.s002.pdf]

S2. Supplementary Results

This section presents supplementary results, focusing specifically on a detailed breakdown of the performance metrics and error patterns observed during the expert validation process, complementing the summary in the main manuscript's evaluation section.

S2.1 Detailed Validation Performance and Error Analysis

The validation results reveal high performance aligned with pharmacovigilance needs: exceptional precision (98.02%; 95% CI: 0.9307–0.9946) and specificity (97.22%; 95% CI: 0.9043–0.9923), indicating the algorithm rarely makes incorrect matches or associates distinct medications. This is crucial for ensuring signal reliability [1, 2, 3]. The moderate recall (77.34%; 95% CI: 0.6936–0.8374), resulting in more false negatives than false positives, reflects a deliberate design choice favouring precision over exhaustive coverage. This conservative approach minimizes potentially misleading false positive signals, aligning with established best practices in safety surveillance where improving the positive predictive value is often prioritized [4, 5, 6]. The algorithm's strong balanced performance (Balanced Accuracy 87.28%, MCC 0.7159) confirms its effectiveness across both matching and non-matching decisions despite dataset imbalance.

Error analysis provided valuable insights. False positives were extremely rare (1.98%), typically involving closely related but distinct drugs. False negatives (29.29% of "NO" decisions), where the algorithm missed true matches, often involved complex synonymy (e.g., Aspirin/Acetylsalicylic acid), regional/international naming variants (e.g., Paracetamol/Acetaminophen), uncaptured abbreviations, or multilingual equivalents. The high inter-rater reliability between experts (κ 0.8405) underscores the inherent difficulty of resolving these cases, which often require specialized domain knowledge beyond simple algorithmic matching. Overall, the human validation confirms the pipeline's high reliability and suitability for pharmacovigilance applications demanding trustworthy standardization.

References

[1] Harpaz R, DuMouchel W, LePendu P, Bauer-Mehren A, Ryan P, Shah NH. Performance of pharmacovigilance signal-detection algorithms for the FDA adverse event reporting system. Clinical Pharmacology & Therapeutics. 2013;93(6):539–546.

[2] Hauben M, Aronson JK. Defining signal and its subtypes in pharmacovigilance based on a systematic review of previous definitions. Drug safety. 2009;32:99–110.

[3] Bate A, Evans SJ. Quantitative signal detection using spontaneous ADR reporting. Pharmacoepidemiology and drug safety. 2009;18(6):427–436.

[4] Fusaroli M, Giunchi V, Battini V, Puligheddu S, Khouri C, Carnovale C, et al. Standardization of drug names in the FDA adverse event reporting system: the DiAna dictionary. medRxiv. 2023; p. 2023–06.

[5] Coloma PM, Trifirò G, Schuemie MJ, Gini R, Herings R, Hippisley-Cox J, et al. Electronic healthcare databases for active drug safety surveillance: is there enough leverage? Pharmacoepidemiology and drug safety. 2012;21(6):611–621.

[6] Hauben M, Bate A. Decision support methods for the detection of adverse events in post-marketing data. Drug discovery today. 2009;14(7-8):343–357.
